# The Mediating Role of Depression and the Moderating Role of Game Genre in the Relationship Between Suicidal Thoughts and Computer Game Addiction Among Adolescents in Tehran

**DOI:** 10.62641/aep.v53i5.1886

**Published:** 2025-10-05

**Authors:** Nima Rezvani, Zahra Jahanbakhshi, Asma Tabatabaeian, Shima Pajouhinia, Sayed Jafar Ahmadi

**Affiliations:** ^1^Department of Counseling, Faculty of Education and Psychology, Shahid Beheshti University, 19839 69411 Tehran, Iran; ^2^Department of Counseling, Faculty of Educational Sciences and Psychology, Shahid Beheshti University, 19839 69411 Tehran, Iran; ^3^Department of Counseling, Faculty of Education and Psychology, Alzahra University, 19938 93973 Tehran, Iran; ^4^Department of Clinical Psychology, Faculty of Psychology and Educational Sciences, Allameh Tabatabaei University, 14896 84511 Tehran, Iran; ^5^Psychology Program, Bard College, Annandale on Hudson, NY 12504, USA; ^6^Department of Psychology, The New School, New York, NY 10011, USA

**Keywords:** computer game addiction, suicidal thoughts, depression, game genre, adolescents

## Abstract

**Background:**

Computer games have become a significant recreational activity for individuals of all ages, particularly children and adolescents. This study aimed to predict suicidal thoughts based on computer game addiction, considering the mediating role of depression and the moderating role of the game genre among adolescents in Tehran.

**Methods:**

Using structural equation modeling, a correlational design was considered for this purpose. The study population included 261 adolescents aged 12–18 years residing in Tehran. Participants completed the Farhadi Computer Game Addiction Questionnaire (2017), Beck’s Suicidal Ideation Scale (1961), and Kutcher Adolescent Depression Scale (2002). The obtained data were analyzed using SPSS-26 and AMOS 24.

**Results:**

The direct paths between suicidal thoughts and computer game addiction (β = 0.17), suicidal thoughts and depression (β = 0.51), and depression and online game addiction (β = 0.70) were found to be significant. The relationship between computer game addiction and suicidal thoughts and the mediating role of depression was not significant in adventure, traditional/educational, and simulation genre groups. However, it was significant in the action genre group (β = 0.32, *p* < 0.05).

**Conclusion:**

These findings underscore the need for targeted interventions by counselors and psychologists to address the negative psychological effects of computer game addiction, particularly among adolescents engaging in action games.

## Introduction

In recent years, computer games have rapidly gained popularity among individuals of various ages and have become an important part of the entertainment of different people, particularly children and adolescents [1]. The continuous improvement in game quality and diversity and advancements in electronics and computing have fueled the growing interest in this form of entertainment, especially among younger demographics [2]. As a result, video games have become a recreational activity for people in the last 50 years [3]. Global statistics indicate that by the end of 2018, there were approximately 2.3 billion gamers worldwide [4]. As engagement in computer games increases, so does the risk of developing an addiction to these games [5]. In computer game addiction, behavioral patterns related to digital gaming involve a preference for gaming over other activities, frequently resulting in weakened willpower and prioritization of games [6]. This addiction has various psychological and social consequences, including increased aggression, anxiety, depression, impaired academic performance, loneliness, and reduced life satisfaction [7, 8].

Another consequence of computer game addiction is the formation of suicidal thoughts, especially among adolescents [9]. Suicide encompasses suicidal thoughts, suicide attempts, and completed suicide, all of which indicate the desire for death. However, these three concepts have important distinctions; suicidal thoughts refer to “thinking about or planning suicide”, whereas suicide attempt refers to “non-lethal, self-directed potentially damaging behavior with the intent to die as a result of this behavior”. In addition, completed suicide signifies death resulting from self-directed damaging behavior with the intent to die [10]. Suicide is the second leading cause of death after unintentional injuries among the 15–19 age groups [11], and the global suicide rate is increasing day by day. For instance, the United States witnessed a 30% increase in suicide rates between 2000 and 2016 [12]. Several studies [9, 13, 14, 15, 16] have examined the relationship between suicide risk and pathological use of computer games. For example, Ivory *et al*. [17] investigated various risk factors associated with gaming frequency among American university students and demonstrated a link between computer games and the formation of suicidal thoughts. Similarly, Byeon *et al*. [18] found that the percentage of suicidal thoughts was higher among individuals who spent hours playing computer games daily compared to those who did not engage in gaming. Among the reasons that may increase the likelihood of suicide in the context of computer games are heightened psychological distress and impulsivity, which, in turn, could elevate suicide risk [19]. Additionally, it is logical to expect that individuals experiencing gaming-related problems may spend more time playing games than other gamers [20]; consequently, they may be more exposed to elements within video games, further enhancing the risk of suicide [21]. However, there is no precise causal link between video game addiction and suicidal thoughts; for instance, Ismail’s study has demonstrated an increase in stress and aggression among individuals who engage in violent video games [22]. Conversely, another study reported a reduction in anger, stress, and aggression among gamers [23].

Another point to consider is that for a more accurate understanding of the relationship between video games and suicidal thoughts, it is necessary to examine the role of a third variable because the association between gaming addiction and suicide may be partially related to common third variables that predict both addiction and suicide (e.g., depression) [19, 20]. Depression is one of the strongest predictors of suicidal thoughts, and the majority (over 90%) of individuals who attempt suicide simultaneously exhibit depressive symptoms. This simultaneous occurrence of depression and suicidal behavior is more than 50% of cases of adolescent suicide [24]. Therefore, investigating the role of depression in the relationship between internet addiction and the formation of suicidal thoughts could play a key role in bridging the causal gap of this connection [25].

As stated by Erevik *et al*. [25], another limitation of existing research lies in the lack of the examination of moderating features of games, including game genres. The genre of a game can significantly impact the addiction level and the formation of suicidal thoughts. Individuals who experience the consequences of computer games may not be uniform in terms of game genre compared to those who do not experience these consequences [26]. Another noteworthy point is that for a more precise understanding of the relationship between playing computer games and the formation of suicidal thoughts, further research is needed among adolescents from different cultural backgrounds, as the existing studies, to the best of our knowledge, have focused on statistical populations of youth [14]. In this context, the present study seeks to explore the relationship between computer game addiction and suicidal thoughts among Iranian adolescents, considering the moderating role of depression and the game genre.

## Methods

This study employed structural equation modeling (SEM). The statistical population of this research consisted of adolescents aged 12–18 living in Tehran province who dedicated more than four hours daily to playing video games. Overall, 261 adolescents participated in this research. The inclusion criteria for their selection included adolescents 12–18 years old, residents of Tehran, and experience of playing computer games for at least 6 months at least 3 days a week. On the other hand, the exclusion criteria were a history of severe psychiatric disorders such as schizophrenia, bipolar disorder, or psychosis according to the report of a psychiatrist, physical or cognitive disability that prevents the completion of questionnaires, and participation in similar studies within the last 6 months.

This study is part of a larger project regarding the factors of suicide. Initially, the researchers obtained permission (with number 1403.183) from the Ethics Committee of Shahid Beheshti University to conduct the research. The statistical population included all teenagers in Tehran. Three popular gaming clubs were chosen from the north, south, and center of Tehran. It should be noted that to comply with ethical principles, the contact numbers of the guardians of teenagers who wanted to participate in the study were taken, and they were contacted; then, the questionnaire link was sent to them along with the consent form. Accordingly, there was direct communication with the participants. These surveys were created on the Porsline website and shared online through links. The participants were requested to follow the link, read the necessary instructions regarding research goals, answer the questions, and allocate sufficient time to respond to all the questions. In the instructions provided to the participants, it was emphasized that they should complete the questions independently and honestly, and it was indicated that no personal information would be collected from them and their identity would be preserved. The data collection phase took place between November 2022 and January 2024.

## Research Tools

### Beck’s Suicide Ideation Questionnaire 

This self-report scale, developed by Aaron T. Beck *et al*. [27] at the University of Pennsylvania in the United States, consists of 19 questions. This questionnaire was used to detect and measure the intensity of attitudes, behaviors, and planning for committing suicide over the past week. The scale was set up based on a three-point Likert-type spectrum ranging from zero to two. The individual’s overall score was calculated by summing the scores, which ranged from zero to thirty-eight. The scale assesses various aspects, including death wishes, active and passive suicidal tendencies, the duration and frequency of suicidal thoughts, feelings of self-control, suicide-inhibiting factors, and the degree of an individual’s readiness for suicide. The questionnaire included five screening questions. If the responses indicated active or passive suicidal tendencies, the participant answered the remaining 14 questions. Based on factor analysis of psychiatric patients, this scale is a combination of three factors, namely, tendency to die (five questions), readiness for suicide (n = 7), and tendency to commit suicide (n = 4). Additionally, two questions pertain to suicide inhibitors or the concealment of suicidal thoughts, which are not included in any of the three aforementioned factors. The results represented concurrent validity with a coefficient of 0.76 and reliability estimated using Cronbach’s alpha of 0.95 for the overall questionnaire score in the present study [27].

### Kutcher Adolescent Depression Scale

This scale, developed by Brooks *et al*. [28] in Canada and at Dalhousie University in Halifax, Nova Scotia, assesses the severity of depressive symptoms in adolescents. It is a self-report instrument consisting of 11 items, and respondents are required to answer each statement based on their recent emotional state over the past week using a 4-point Likert-type scale. Scoring on this scale is direct, and the total score for an individual can range from 0 to 33. Additionally, this assessment includes two subscales, namely, major depression and suicidal thoughts. In the current study, internal consistency reliability was established using Cronbach’s alpha, resulting in a reliability coefficient of 0.89 for the overall depression score.

### Farhadi Computer Game Addiction Questionnaire

This questionnaire, developed in Iran and Islamic Azad University of Isfahan (Khorasgan) [29], consists of 19 items. All items in this tool are scored on a 5-point Likert-type scale (4 = Always, 3 = Often, 2 = Sometimes, 1 = Rarely, and 0 = Never), and the score range in this questionnaire is between 0 and 52. A higher score indicates greater addiction to computer games and vice versa. In the study [29], to assess the questionnaire’s validity, it was initially administered to 98 or more participants, and the split-half method and Cronbach’s alpha were used. After splitting the questionnaire items related to computer game addiction and calculating the scores for each half, the correlation coefficient between the scores obtained from the split-half method was 0.77, and a reliability coefficient of 0.87 was obtained using the Spearman-Brown method. Furthermore, the reliability coefficient obtained using the internal consistency method (Cronbach’s alpha) was 0.90, indicating high reliability. In the current study, internal consistency reliability was established using Cronbach’s alpha, resulting in a reliability coefficient of 0.93 for the overall computer game addiction score.

### Data Analyses

To check the study’s hypotheses, a structural model was designed in the form of total scores of all four variables. AMOS is one of the most successful statistical software programs, especially designed for SEM. This software is marketed by IBM (it is an American multinational technology company based in Armonk, New York, USA), the manufacturer of SPSS software. The data were analyzed using SEM and confirmatory factor analysis, which was performed through SPSS (version 26) and AMOS (version 24, Armonk, New York, USA). After confirming the confirmatory factor analysis of the variables, first, direct path coefficients between the criterion variable (suicidal thoughts) and the predictor (computer game addiction), as well as indirect coefficients with depression as a mediator, were examined to determine the mediating variable. Subsequently, the moderating role of the game genre was investigated using a multigroup approach.

## Results

The results revealed that out of 261 adolescents within the age range of 12–18 years, the average age (±standard deviation) was 15.92 (±1.92).

Regarding examining the three assumptions for the structural modeling analysis, the data distribution was normal, and there were no outliers or missing data.

The results (Table 1) demonstrated that the data distribution was within the normal range of skewness (±2) and kurtosis (±3). Discriminant validity specifically measures whether constructs that theoretically should not be related to each other are, in fact, unrelated. According to the accepted statistical rule, a correlation less than 0.9 shows the discriminant validity of two variables. The correlation coefficient between the variables is significant and less than 0.9, indicating the discriminant validity of the variables (Table 1).

**Table 1.** **Mean, standard deviation, skewness, kurtosis, and bivariate correlation between variables**.

Variable	Mean	SD	Sk	Ku	Correlation		
					Suicidal thoughts	Computer game addiction	Depression
Suicidal thoughts	8.72	10.95	1.15	0.17	1		
Computer game addiction	48.09	17.08	0.003	–0.52	0.52**	1	
Depression	11.76	8.14	0.26	–0.59	0.60**	0.64**	1

*Note*. **: Correlation is significant at the 0.01 level (2-tailed). SD, Standard deviation; Sk, Skewness; Ku, Kurtosis.

### Structural Equation Modeling Analysis

**Fig. 1.**
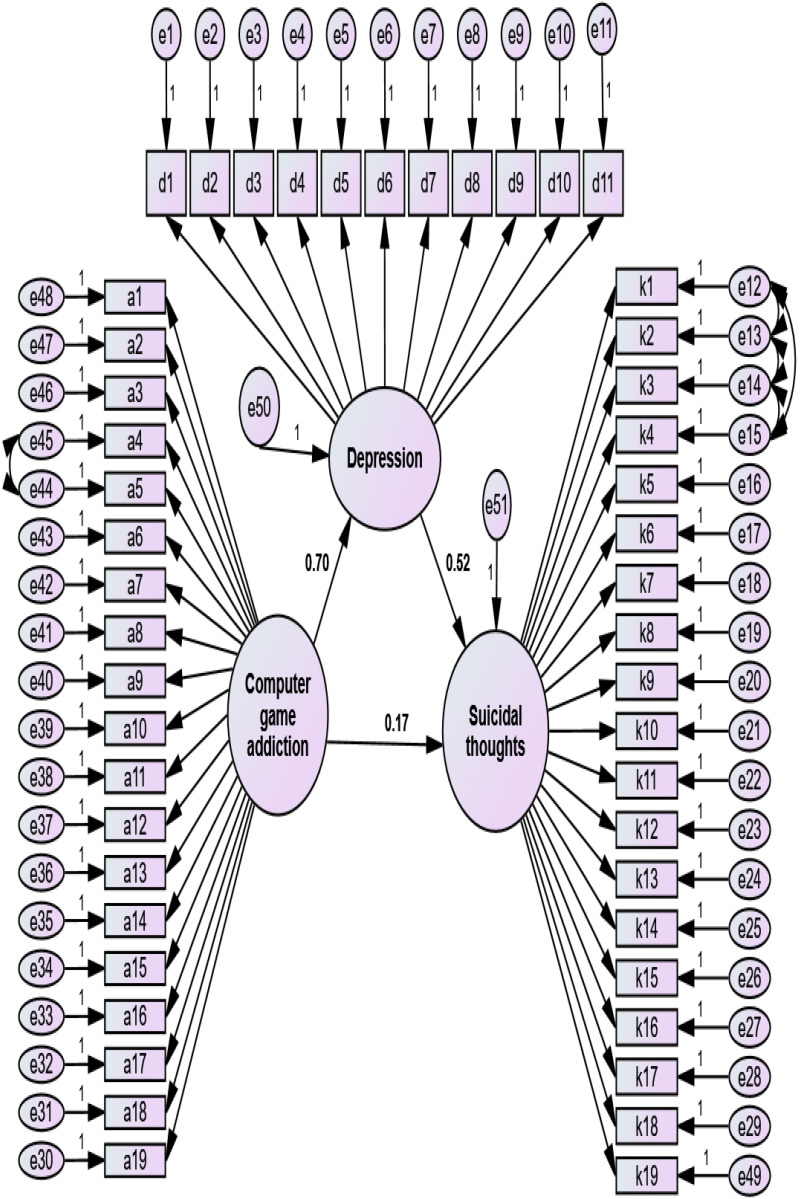
**The mediating model**. *Note*. Path coefficients between computer game addiction and depression (β = 0.70, *p* < 0.05), depression and suicidal thoughts (β = 0.52, *p* < 0.05), and computer game addiction and suicidal thoughts (β = 0.17, *p* < 0.05) were obtained. d1–d11 are depression items, a1–a19 are computer game addiction items, k1–k19 are suicidal thoughts items, e1–e51 are item errors, and two endogenous variables.

In the first stage of evaluating the research variables, all factor loadings for the research items were greater than 0.3, and no item was excluded from the analysis [30]. The mediation model, in which computer game addiction (the predictor variable), depression (the mediator variable), and suicidal thoughts (the criterion variable) were included, is as follows (Fig. 1).

In the second stage, first, the overall fit of the mediation model was examined, and after confirmation, the path coefficients and significance between the research variables in the depression mediation model underwent investigation.

The model was confirmed if at least three indicators met the acceptable fit criteria. Based on the findings, the fit indices were favorable, and the mediation model of depression in the relationship between computer game addiction and suicidal thoughts was confirmed accordingly (Table 2).

**Table 2.** **Fitness indicates**.

Indicators	CFI	GFI	TLI	CMIN/DF	RMSEA
Value	0.91	0.77	0.91	1.87	0.05
Acceptable	>0.9	>0.9	>0.9	<5	>0.03<0.08

*Note*. CFI, Comparative fit index; GFI, Goodness of fit index; TLI, Tucker–Lewis index; RMSEA, Root mean square error of approximation; CMIN/DF, Minimum discrepancy of confirmatory factor analysis/degrees of freedom.

The direct paths between suicidal thoughts and computer game addiction (β = 0.17, *p* < 0.05), suicidal thoughts and depression (β = 0.52, *p* < 0.05), and depression and online game addiction (β = 0.70, *p* < 0.05) were significant. The mediation model of depression in the relationship between suicidal thoughts and computer game addiction was confirmed based on the results (Table 3).

**Table 3.** **Path coefficients**.

Path coefficients		Direct effect		Indirect effect		Total effect	
		β	*p*	β	*p*	β	*p*
Suicidal thoughts	Computer game addiction	0.17	0.03	0.36	0.001	0.54	0.001
Suicidal thoughts	Depression	0.52	0.001	-	-	0.52	0.001
Depression	Computer game addiction	0.70	0.001	-	-	0.68	0.001

### Moderating Role of Game Genre

The final stage examined the moderating role of the game genre in the relationship between computer game addiction and suicidal thoughts, with depression as a mediator. The participants were categorized into four game genres, namely, adventure (n = 60), action (n = 67), simulation (n = 76), and traditional/educational (n = 58). If the fit indices for the variable model are better than those for the fixed model, it can be concluded that the genre variable plays a moderating role.

The results confirmed that the fit indices for the variable model were better than those of the fixed model, suggesting a moderating role of game genre in the assumed model (Table 4).

**Table 4.** **Models’ fitness indices**.

Indicate	CMIN/DF	GFI	CFI	IFI	RMSEA
Unconstrained	2.28	0.48	0.60	0.60	0.06
Measurement residuals	2.27	0.47	0.57	0.57	0.07

*Note*. CFI, Comparative fit index; GFI, Goodness of fit index; IFI, Increasing fit index; RMSEA, Root mean square error of approximation; CMIN/DF, Minimum discrepancy of confirmatory factor analysis/degrees of freedom.

Next, the beta coefficients for predicting suicidal thoughts across the four game genre groups were examined.

Based on the obtained data (Table 5), the relationship between computer game addiction and suicidal thoughts and the mediating role of depression were not significant in the adventure (β = 0.11, *p* > 0.05), traditional/educational (β = 0.28, *p* > 0.05), and simulation (β = 0.11, *p* > 0.05) genre groups. However, it was significant in the action genre group (β = 0.32, *p* < 0.05). Therefore, the results confirmed the moderating role of game genre in the relationship between suicidal thoughts based on the computer game with the mediating role of depression.

**Table 5.** **Moderating role of the game genre**.

Variable independent	Variable dependent	Adventure genre		Action genre		Simulation genre		Traditional genre	
		β	*p*	β	*p*	β	*p*	β	*p*
Computer game addiction	Suicidal thoughts	0.11	0.32	0.32	0.04	0.11	0.44	0.28	0.13

## Discussion

The results revealed that computer game addiction and depression are directly associated with suicidal thoughts. Furthermore, a structural equation model demonstrated that depression acts as a mediator in this relationship, implying that depression partially mediates the impact of computer game addiction on suicidal thoughts. Additionally, this study examined the moderating role of game genre. The results showed that the relationship between computer game addiction and suicidal thoughts was moderated in the action genre. These findings suggest that game type plays a crucial role in moderating the effect of game addiction on suicidal thoughts. These results are in line with those of previous research [14, 31, 32, 33], indicating that computer game addiction increases the risk of suicide.

Exploring the relationship between computer game addiction and the formation of suicidal thoughts in adolescents based on conflict theory, it can be asserted that computer games are associated with various conflicts among adolescents [34]. These conflicts include the tension between reality and fantasy, concentration and dispersion, social and individual needs, and the real versus ideal self, which may lead to the formation of suicidal thoughts in adolescents. Game genres can significantly contribute to these conflicts, as some genres address specific needs, providing individuals with a sense of control and satisfaction, whereas others may exacerbate feelings of stress and helplessness. Certain genres, such as violent games and those closely tied to reality, can intensify tension and the inability to escape, potentially accelerating the formation of suicidal thoughts [21]. Other genres can also serve as positive moderators. For example, adventure games can enhance a person’s sense of imagination and creativity, serving as an escape from reality; however, they are generally less associated with suicidal thoughts. Furthermore, Koga *et al*. [14] demonstrated that individuals experiencing depression are more likely to be involved in computer game addiction, and the action game genre also exacerbates depression, while depression is one of the strongest predictors of suicidal thoughts [35].

The results of this study emphasize that computer game addiction may be associated with depression in adolescents, and this depression serves as a mediating variable in the impact of addiction on suicidal thoughts. This important finding not only underscores the importance of paying attention to the psychological consequences of computer game addiction but also highlights its interference with the mental health of young people. Furthermore, the results indicated that the action game genre also plays a significant role in the relationship between computer game addiction, depression, and suicidal thoughts. Within the action game genre, the effect of addiction on suicidal ideation was moderate, highlighting that different types of games may have varying effects on the psychological consequences of computer game addiction.

The participants were collected from a specific region (Tehran County), and due to the need for direct communication with individual respondents, researchers lacked the necessary financial resources to travel to other cities. This may have restricted the generalizability of our results to other areas. To enhance the reliability of the results, future research should focus on collecting samples from various regions. Additionally, a self-report tool was used in this study, whereas employing other methods, such as face-to-face interviews, could improve the accuracy and validity of the results. Another important point is that the current study was conducted cross-sectionally, implying that causal relationships between variables could not be established with certainty. To investigate causal relationships, longitudinal study designs may be appropriate. Additionally, only certain variables were examined in this study. Other factors, such as economic status, cultural background, and other psychological variables, may play a significant role in the relationship between computer game addiction and suicidal thoughts. Further research should be conducted in this area, especially considering the role of gender in this context.

## Conclusion

In general, the findings of this study underscore the importance of computer game addiction in conjunction with the mediating role of depression and the moderating role of the action game genre. It is hoped that these findings will guide individuals involved in youth communities, including parents and counselors, to choose the best strategies for the prevention and management of these psychological issues in adolescent environments. Therefore, awareness of the potential consequences of computer game addiction and its effects on the formation of suicidal thoughts may provide more effective preventive and therapeutic measures for responsible individuals. Moreover, the findings can assist counselors, psychologists, and experts in the field of virtual addiction to adopt the best strategies and interventions to prevent and manage the negative effects of computer game addiction in the adolescent community.

## Availability of Data and Materials

The datasets used and/or analyzed during the current study are available from the corresponding authors on reasonable request.
